# Impact of Fatigue in Rheumatic Diseases in the Work Environment: A Qualitative Study

**DOI:** 10.3390/ijerph121113807

**Published:** 2015-10-28

**Authors:** Deirdre Connolly, Clodagh Fitzpatrick, Lynn O’Toole, Michele Doran, Finbar O’Shea

**Affiliations:** 1 Discipline of Occupational Therapy, School of Medicine, Trinity Centre for Health Sciences, St. James Hospital, James St., Dublin 8, Ireland; E-Mails: fitzpacl@tcd.ie (C.F.); otoolely@tcd.ie (L.O’T.); 2 Department of Rheumatology, St. James’s Hospital, Dublin 8, Ireland; E-Mails: mfdoran@stjames.ie (M.D.); Foshea@stjames.ie (F.O’S.)

**Keywords:** fatigue, rheumatic diseases, disclosure, workplace accommodations, education

## Abstract

Fatigue is a symptom of arthritis that causes difficulty at work. An improved understanding of this symptom could assist its management in the work environment. The aim of this study was to explore people with rheumatic diseases’ experiences of fatigue in work. A qualitative descriptive design was used with semi-structured interviews and a constant comparative method of data analysis. There were 18 participants, the majority of them female with Rheumatoid Arthritis (RA) and working full-time. Three themes were identified: “Impact of fatigue on work performance” with cognition, mood and physical abilities being the main difficulties reported. In the second theme “Disclosure at Work” participants discussed disclosing their disease to employers but reported a lack of understanding of fatigue from colleagues. The final theme “work-based fatigue management strategies” included cognitive strategies and energy management techniques, which were mainly self-taught. In this study, fatigue was reported to impact on many areas of work performance with limited understanding from colleagues and employers. Interventions from health professionals to assist with development of work-related self-management skills are required to assist with symptom management in the work place. Such interventions should include education to employers and colleagues on the nature of fatigue in Rheumatic diseases.

## 1. Introduction 

The term rheumatic diseases covers over 100 diseases such as Rheumatoid Arthritis (RA), and Osteoarthritis (OA) and other auto-immune conditions such as Ankylosing Spondylitis (AS), and Spondyloarthropies, Systemic Lupus Erythematosus (SLE), Fibromyalgia and Systemic Sclerosis (SSc) [1]. A worldwide report on the prevalence of arthritis and rheumatism reported the prevalence of OA between 8% and 16.4%, RA between 1% and 6%, AS between 0.1% and 0.5% and autoimmune conditions such as SLE, SSc and Sjögren’s Syndrome as between 0.1% and 0.5% [2]. In Ireland, approximately 915,000 people have a Rheumatic disease with RA, OA and Fibromyalgia being the most common [3]. It has been reported that rheumatic diseases are one of the main causes of physical disability, contributing to societal and economic costs including loss of productivity in the workplace [4]. 

Work disability in rheumatic arthritis is considered a multidimensional concept that encompasses more than just employment status. It includes forced reduction in employed hours, loss of prospects of being promoted, more frequent use of sick leave, and early retirement [5]. Rheumatic diseases are one of the most common types of chronic conditions to affect a person’s ability to remain in paid employment [6]. For example, a Dutch study reported that people with AS are three times more likely to withdraw from work than the general population [7]. Three main challenges in the work place have been identified. The first relates to the impact of symptoms such as fatigue and pain on productivity levels and absenteeism. The second challenge relates to the work environment including issues with colleagues. The final challenge identified arises from the emotional impact of work disability on the person with the rheumatic disease [8,9].

Due to its high prevalence in people with rheumatic diseases, Gignac *et al.* maintain that fatigue must be considered in any study of work disability and productivity [10]. A study by De Croon *et al.* [11] on work ability of 78 employees with early Rheumatoid Arthritis (RA) found that predictors of low work ability include fatigue, use of manual strength at work, and a lack of support, autonomy and participation, in decision making. A qualitative study [12] identified fatigue as a symptom of rheumatoid arthritis that caused most difficulty at work. Participants also identified a lack of understanding of fatigue by employers and colleagues due to it being an invisible symptom. Although this study identified fatigue as impacting work ability, it did not investigate specific work-related difficulties caused by fatigue. Most studies limit their inclusion criteria to a single diagnosis of rheumatic diseases, whereas the current study includes diagnoses of all rheumatic diseases.

Although fatigue causes difficulty for people with rheumatic diseases, many report that they do not discuss this symptom during clinic appointments because it is not acknowledged by health professionals [13]. Therefore, in order to assist health professionals address this symptom with their patients regardless of their type of rheumatic disease, there is a need to identify the specific challenges people with fatigue experience in the work place and what strategies facilitate work ability and performance [12,13]. 

Therefore the aims of this study were:

1. To explore experiences of the impact of fatigue on work ability of people with Rheumatic diseases.
2. To identify how fatigue impacts on work tasks of people with Rheumatic diseases.
3. To investigate the range of strategies used in the work place to manage fatigue.

## 2. Methods

### 2.1. Design

This study was part of a larger mixed methods study investigating the impact of work ability for people with Rheumatic diseases. The qualitative phase (reported here) employed a Qualitative Descriptive (QD) approach as described by Sandelowski [14] and was guided by the Consolidated Criteria for Reporting Qualitative Research (COREQ) framework for designing and reporting qualitative studies [15]. QD is a qualitative research design used to describe peoples’ perceptions and experiences of a particular phenomenon. It differs from other qualitative methods in that data is collected through semi-structured interview and data analysis is descriptive rather than interpretive [16]. QD is considered an appropriate methodology for needs assessments and for informing planning and delivery of clinical interventions [16]. Neergard *et al*. [17] also state that it is widely used by healthcare professionals and is recommended for small scale, independent, research projects. 

#### Participants and Procedures

Participants for the larger study were recruited through an out-patient Rheumatology clinic in a large teaching hospital. The inclusion criteria for that study were that participants were between the ages of 18–65, had a definite diagnosis of a rheumatic disease (as confirmed through medical chart review), and were currently working. All study participants completed a number of questionnaires related to fatigue, work-role functioning, arthritis-work spillover and quality of life. At the end of the questionnaires, participants were asked to provide their contact details if they were willing to participate in an interview to discuss the impact of fatigue on their work performance. Of the 234 who completed the questionnaires, 43 people agreed to be interviewed; however, when contacted to arrange the interview, many either declined or failed to return the researchers’ telephone calls. The final number, therefore, that participated in the interviews was eighteen people. On the day of interview, written consent to participate was received from all participants.

### 2.2. Data Collection

**Figure 1:**
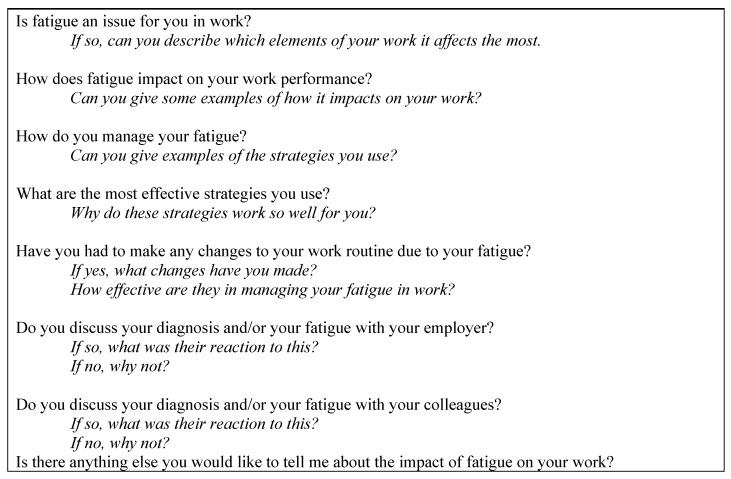
Interview questions and probes (in italics).

Interviews were carried out over a four-month period by one researcher (CF) in a quiet room in the university where the study was carried out. A semi-structured interview guide (Figure 1) was used to focus the discussion so that it was in line with the study aims while also seeking people’s personal experiences [18]. Probe questions were used to gather more specific details on the impact of fatigue in the workplace (Figure 1). The interview guide was posted or emailed to participants before the interview to enable them to prepare for their interview. Interviews lasted between 15 and 70 min.

Before the interview commenced, all participants were provided with written and verbal information about the study aims and written and verbal consent was obtained. Ethical approval was received for the study from the recruiting hospital research ethics committee prior to commencement of the study. 

### 2.3. Data Analysis

All interviews were audio recorded and transcribed verbatim. Each participant was sent a copy of their interview transcript to enable them to remove or amend any information given during their interview. None of the participants made any changes to their transcripts.

Data analysis began with three researchers (Deirdre Connolly, Clodagh Fitzpatrick and Lynn O’Toole) coding a single interview. Coding is the practice of grouping and labelling ideas and views so that themes are generated that reflect wider perceptions [18]. Quotes that stood out as significant and related to the study aims were highlighted and coded as suggested by Layder [19]. Following the independent coding process, differences in codes were discussed until agreement could be reached or codes re-named. The remaining seventeen interviews were then allocated among the three researchers and all agreed codes were applied to these interviews. As new codes were identified, they were shared among the three researchers and applied as appropriate. On completion of the manual coding process, transcripts were entered into a qualitative data management software programme Nudist Vivo 10 (NVivo) and all codes were grouped into categories. These categories were then labelled as themes to reflect the codes within each category and according to their relevance to the research objectives. In QD, identified themes clearly reflect the aims of the study [17]. Three themes relating to fatigue in the workplace were identified: impact of fatigue on work demands; disclosure of disease and fatigue; and work-based fatigue management strategies. 

## 3. Findings

The majority of participants were female (*n* = 12), between the ages of 31–40 years (*n* = 8) and married (*n* = 8). Eleven participants were working full-time and the majority was in non-manual type work. Of those who supplied information on their employers, six people worked for private companies or were self-employed, and six people worked in Public Sector/Government positions. See Table 1 for work characteristics of each participant. 

**Table 1:** Work characteristics of participants.

Participant ID (Gender)	Age Category (Years)	Diagnosis ^≠^	Work Type *	Work Sector/Employer	Work Hours
P1 (M)	31–40	AS	Manual	Missing	Part-time
P2 (F)	51–60	RA	Mixed	Private	Part-time
P3 (F)	31–40	PsA	Non-manual	Public sector/Government	Part-time
P4 (M)	41–50	AS	Manual	Missing	Full-time
P5 (M)	31–40	Other	Manual	Missing	Full-time
P6 (F)	41–50	PsA	Manual	Missing	Full-time
P7 (F)	31–40	RA	Non-manual	Public sector/Government	Full-time
P8 (F)	18–30	RA	Non-manual	Public sector/Government	Full-time
P9 (M)	51–60	RA	Mixed	Private	Part-time
P10 (M)	61–65	RA	Non-manual	Public sector	Part-time
P11 (F)	18–30	Other	Mixed	Missing	Full-time
P12 (F)	31–40	Other	Non-manual	Public sector/Government	Full-time
P13 (F)	31–40	RA	Non-manual	Private	Part-time
P14 (F)	41–50	SLE	Non-manual	Missing	Full-time
P15 (F)	31–40	SLE	Non-manual	Public sector/Government	Part-time
P16 (F)	31–40	SSc	Non-manual	Private	Full-time
P17 (M)	41–50	PsA	Manual	Private	Full-time
P18 (F)	51–60	RA	Non-manual	Private	Full-time

**^≠^** AS: Ankylosing Spondylitis; RA: Rheumatoid Arthritis; PsA: Psoriatic Arthritis; SLE: Systemic Lupus Erythematosus; SSc: Systemic Sclerosis; Other: non specified; * Work type: Manual with no supervisory duties (e.g., construction worker, carpenter, roofer, gardener); Non-manual (e.g., administrative, managerial, supervisory, office and other professional, such as teacher); Mixed non-manual and manual (e.g., sales and service occupations such as waitress, personal care attendant, patient care nurse, nurse’s aide, driver).

Between them, participants had five different types of rheumatic diseases with the most common being Rheumatoid Arthritis (*n* = 7), followed by Psoriatic Arthritis (*n* = 3). There were equal numbers of participants for time since diagnosis in the categories of “less than 5 years” and “6 to 10 years”, with seven participants in each. The remaining participants had their diagnoses over 10 years. Disease activity was measured on a global rating of 1–10 as described by Anderson *et al*. [20]. The mean rating was 4.9 (SD 2), with a range of 2–8. The mean fatigue score on the Multidimensional Fatigue Scale (MFS, [21]) was 14.3 (SD 4.3) with a range of 4–19. Scores on the MFS range from a minimum of 4 to a maximum of 20 with higher scores indicating higher levels of fatigue. A score of 13 or above indicates severe fatigue [22]. 

Qualitative data analysis resulted in three themes: impact of fatigue on work demands; disclosure of disease and fatigue; work-based fatigue management strategies. See Table 2 for themes and sub-themes. 

**Table 2:** Themes and subthemes from qualitative data analysis.

Theme	Subtheme
Impact of fatigue on work demands	Impact on cognition Impact on mood Impact on physical abilities
Disclosure of disease and fatigue	Reasons for telling or not telling Reactions of employers and colleagues Supports needed for disclosing fatigue in work
Work-based fatigue management strategies	Cognitive strategies Energy conservation strategies

Fatigue was described by all participants as having an impact on their ability to work. All participants experienced considerable fatigue “*I wake up tired every day*” (P5) and “*For me the fatigue is an absolute killer*” (P6). Some participants described an unpredictability of fatigue. For example, “… *it suddenly comes upon you. I’d be sitting at my desk and literally nodding off. It just comes out of the blue*” (P11).

### 3.1. Impact of Fatigue on Work Demands 

Participants reported three areas on which fatigue impacted and identified how this interfered with different aspects of their work. 

#### 3.1.1. Impact on Cognition

Mental fatigue and its impact on work was discussed by 16 of the participants: “*the mental fatigue side of it–that would really impact work*” (P9). One participant stated that when he experienced cognitive fatigue in work it felt like his head was “*muzzy*” (P4). Another participant, working at a desk-based job, referred to mental fatigue as “*brain fog*” (P12) when trying to think or concentrate when fatigued. Another participant reported that cognitive fatigue resulted in working at a slower pace because “*I’m just shut down in my head*” (P6). This lack of concentration resulted in making more mistakes: “*the more mentally fatigued I get, the more mistakes I make*” (P16). Mistakes resulted in participants “*doubting*” (P1) themselves which lead to “*double-checking*” (P18 who works in administration and accounts) and re-doing work tasks already completed “… *and then I find myself having to go back to the beginning*” (P3).

“*If I’m reading something and I’m meant to be commenting on it, and then I’m three pages in, I’ve read it all, I’ve read every single word and I haven’t got a clue what they’ve said. So it’s that sort of engagement that your brain sort of just switches off quicker.*”

(P14)

One participant, who described her job as involving both manual and non-manual tasks, described a lack of concentration when talking or interacting with work colleagues: “*In meetings sometimes I find it very hard to concentrate and to keep listening. I’ve had people say to me ‘are you listening to me?’*” (P2)

Difficulty with memory was also identified as a result of being fatigued: “Tiredness makes me very forgetful. I’d have to have my list—“this is what I’m going to do today!” (P4) and “Just walking into a room to do something and thinking “what did I come in here to do?” (P11). Other memory difficulties which impacted work performance included difficulty remembering words, colleagues’ names, phone numbers and passwords:

“*I can’t remember people’s names or phone numbers. Like the password for my computer, I have it for years. And yet there’ll be times I’ll go blank and think ‘what the heck is it?’. And it’s just when I’m tired, it’s pure tiredness*”

(P8)

#### 3.1.2. Impact on Mood

Some participants gave examples of how fatigue made them irritable in work “*Your tolerance levels drop because you’re tired*” (P9, customer support for a privately owned company). Another participant described an impact on his relationship with his colleagues or customers as a result of being irritable:

“*When I’m really tired, it makes it more difficult for me to just ignore when people get a bit angry, or rant at you. It’s easier for me to take it personally when I’m already in a bad mood.*”

(P14)

Participants also discussed other emotions they experienced in work as a result of difficulties encountered because of fatigue. These included guilt “*I used to feel really guilty when I went for a nap because I would think that I’m being really lazy. But now I know that I actually need it*” (P15); feeling depressed “*I try to remain upbeat but there are days that I would be down, purely because I'm just exhausted.*” (P8); reduced motivation with respect to work “*when I'm that tired I just don’t care. Even when people are talking to you it’s just* ‘*go away’*” (P4) and a changed attitude to work:

“*I’ve come to hate my work, I really have come to hate it. I just don’t like it anymore. I’d rather be licking stamps and putting them on envelopes than actually having to go in and push myself as hard as I have to push myself just to get through the day*”

(P5, construction worker)

#### 3.1.3. Impact on Physical Abilities

Participants described a physical fatigue they experience in work: “*I feel like I’m shaking inside but I look down at my hands and legs and they’re not shaking*” (P15)

“*I feel like there’s porridge in my veins instead of blood. Everything is heavier and slower—and you’re constantly dragging yourself around- so I call it porridge blood.*”

(P3)

Another participant discussed how sometimes the physical fatigue can lead to absenteeism: “I might get up in the morning and I won’t be going anywhere because physically I won’t be able to walk—I’m just too tired.” (P10) 

Participants spoke about trying to avoid work activities or tasks that involve physical activities such as going outdoors, moving objects such as computers and lighter objects such as boxes of paper “*There would be carrying and lifting but I try to minimise it as much as possible. Like, get somebody to do it for me or use a trolley*” (P2)*.* Two participants (P11 and P16) spoke of always having to take a short rest after using the stairs in their workplace. This lead to feelings of embarrassment as they believed their colleagues lacked understanding: “*You know they just don’t understand*” (P16).

### 3.2. Disclosure of Disease and Fatigue

#### 3.2.1. Reasons for Telling or Not Telling

Fifteen participants had disclosed to their employer about their rheumatic condition “*I’ve been open from day one. I think if I’m open with them, they can be open with me*” (P8). Some participants reported being uncomfortable telling their employers about their arthritis but felt it was necessary to explain frequent absences from work. Only three participants had not told their employers about their rheumatic disease. When asked why not, they gave a number of reasons such as being on a probation period, afraid of being made redundant upon disclosure, fear of being observed constantly and a lack of understanding, “*But I wouldn’t tell my boss something like that because you’re afraid. What if they think that you’re just not able for it and you shouldn’t be there*” (P14).

Another participant who had just started a new job was on probation and would not disclose to his employer: “I’m on a probation period, and I’d be afraid that they would say “well, somewhere along the line this fella is probably going to be taking more and more time off, so what’s the point in holding on to him?” So, I just won’t tell them” (P17).

This participant believed that the issue of disclosure related to who the employer is: “It also depends on what type of job you have. Maybe if you were working for the Government, it’d be easier to tell them. But most employers really don’t care. They just want you to get on with it and make them money, and that’s all they want to know about” (P17).

#### 3.2.2. Reactions of Employers and Colleagues

The majority of participants reported that their employers were supportive of the physical aspects of their disease and offered them appropriate accommodations such as ergonomic assessments:

“*And they got in an ergonomic specialist to make sure that everything was within reaching distance for me so that I wasn’t over stretching and everything was in the right place.*”

(P12)

However, some participants discussed a lack of understanding of fatigue from employers and colleagues when disclosing their fatigue. This lead to difficulties for participants in explaining their fatigue to their colleagues: “*They don’t understand the scale of the tiredness. A lot of people don’t realize—nobody really gets it*” (P8). Some participants reported varied responses with regards to support from their managers:

“*It was mixed between different people. Like, the manager that would be over my area, he was like- ‘how can we help you?’- But I don’t find the person I report directly to as positive or as helpful*”

(P2)

This lack of understanding had led to confusion for participants on what accommodations are reasonable for employers to provide with regards to managing fatigue at work:

“*If you had a headache you could go in and say ‘oh I’m really suffering with a bad headache now, I need to go home.’ But you can’t go in and say ‘I’m really, really tired, I need to go home.’*”

(P15)

Some participants were offered reduced hours or flexi-time which helped them in managing their fatigue. This demonstrates that some employers are providing accommodations to people with rheumatic conditions. The participants whose employers and colleagues were supportive reported feeling reassured by the fact that there was awareness of their health condition in the workplace “*But the fact that work are so good, and that they know about my condition, I feel I don’t have to pretend I’m okay when I’m not okay*” (P9). 

Two participants (P4 and P5) working in manual jobs believed that their employers were more interested in the work being completed than in their employees’ health: “*Even though they would know* (of the diagnosis), *they’d say ‘this job is not getting done here—what’s the story?*’” (P4). 

#### 3.2.3. Support Needed for Disclosing Fatigue in Work

Study participants discussed how best to inform employers and colleagues about fatigue and the impact of their rheumatic disease on their work. One participant, currently working in a government-funded position, stated that because she had informed a previous employer that it made it easier to disclose to her current employer:

*I wouldn’t have told people in my previous job only things got really bad. And it was that experience of it going down so well that then allowed me to be just completely open about it when I came here*”

(P7)

Some participants spoke about the role of health professionals in coaching people with arthritis on how to inform their employers and colleagues about their rheumatic disease and their fatigue. One suggestion was to bring a health professional to meetings of disclosure with employers:

“*But also there’s a bit of work there with us and thinking about how you tell people. So there’s almost giving us permission and thinking about what you can do with people with arthritis themselves, to empower them to say* “*I’m actually going to do this*”*. I’m going to take this information or I’m going to encourage my OT (Occupational Therapist) to come with me to tell my employer.*”

(P3)

Other suggestions for educating employers and colleagues included providing written and verbal information about fatigue in rheumatic conditions to increase their understanding: “*Education sessions explaining about fatigue and how it can affect employees and what employers could possibly do to help them.*” (P18)

Some discussion occurred around the health professionals’ awareness of fatigue and its management: “*Nobody says ‘what are you doing for the fatigue?’ The health professionals don’t understand how to treat it either*” (P15). Participants believed that sometimes health professionals did not recognise fatigue as a symptom and that further education amongst health professionals was needed. 

### 3.3. Work-Based Fatigue Management Strategies 

Participants discussed a range of strategies for managing and coping with fatigue in the workplace. These involved cognitive strategies and energy management techniques.

#### 3.3.1. Cognitive Strategies

Some participants discussed using cognitive-based strategies such as changing from a negative to positive attitude to help them get through the working day “*So I made a conscious decision to stop it and to make more of an effort myself and that makes it more bearable*” (P13). Another participant reported: “*you just have to get on with it. You just work around it*” (P7). 

#### 3.3.2. Energy Management Techniques

Participants identified a range of energy management strategies they used to manage their fatigue over the working day and week. These included pacing, planning and prioritising work tasks. 

#### 3.3.3. Pacing 

One participant who works in construction discussed pacing himself between the physical aspects of his work and administrative tasks: “*I try to not do the lifting things when I’m really tired. Those days I’ll do the office work. I try to organise it so that I do the tiling every few days instead of every day*” (P9). Another technique described was changing tasks when concentration is waning “*Just do a paragraph, just focus on that paragraph, is there anything there? Stop, do something else and come back to it*” (P3). One participant described a negative consequence of pushing herself to complete her work activities:

“*So when I am able to work, I work as long as I can. I’d go in at eight in the morning and stay ‘til seven or eight in the evening. And I’d keep on pushing through. Come Sunday I’m zonked—absolutely zonked after five days of doing that.*”

(P12)

#### 3.3.4. Resting

The majority of participants discussed the importance of taking rest and short breaks between tasks. They described taking all scheduled breaks such as lunch breaks and also taking short movement breaks such as getting up from their desk to get a glass of water or go to the bathroom. The benefit of short rests during the work day was identified by fifteen participants. For example: “*ten minute breaks are great*” (P4) and “I *go into the cubicle and stay there for ten minutes and I’ll just sit there and I’ll probably have a quick doze and that helps*” (P8).

#### 3.3.5. Planning 

Planning ahead was another energy conservation strategy that participants identified as being beneficial for managing their fatigue in work. Participants spoke about planning and organising their work routine according to their fatigue levels:

“*I work full time and by Thursday evening I’m not worth tuppence. So, I always plan to keep Friday as quiet and as calm as I can because I know I’m not going to function well on Friday.*”

(P8)

One participant identified planning a rest day in-between working days as a useful strategy: “*I found that planning a rest day helps.*” (P15).

Strategies were also discussed in relation to reducing working hours or working flexi-time due to fatigue levels. Six participants had changed from full-time to part-time work. One participant organised her work schedule to ensure she only works a maximum of three days consecutively:

“*I’ve gone job sharing this year. It’s a split week so it’s Thursday, Friday and then the weekend and then Monday, Tuesday Wednesday. So I never work more than three days in a row*”

(P15)

#### 3.3.6. Prioritising 

Participants also discussed other organisational strategies for managing their workload such as making lists, organising emails, delegating tasks and spreading tasks throughout the week instead of doing them all in one day:

“*So I go in and I’ve got my list and I just work through it. So it’s being organised and just keeping going—not too fast. As you say, taking it easy but keeping moving on things.*”

(P3)

## 4. Discussion

### 4.1. Impact of Fatigue in Work

Eighteen people participated in this study. The majority were female, worked full-time and had Rheumatoid Arthritis. There were five different types of rheumatic disease between the eighteen participants. All participants reported that fatigue was the symptom that impacted most on their work ability. Other research of employed adults with RA also found that participants viewed fatigue as the aspect of their condition most limiting their employment and impacting on many aspects of work functioning [8,13]. Addressing fatigue through interventions and appropriate accommodations by health professionals and employers may help to validate experiences of fatigue, improve self-management of symptoms and enhance work functioning. It may also be important to target those who experience fatigue early post diagnosis to facilitate development of strategies that can be used in response to variations in the course of a disease such as during a relapse.

Participants discussed the impact of fatigue on their cognition, mood and physical abilities within the workplace and provided specific examples of work-based tasks affected by this. They also described how these difficulties often impacted on their interactions with colleagues. Interventions are therefore required to specifically address these three areas and how to minimise their impact on work activities. Empowering individuals to manage their fatigue and its emotional impact may be needed for individuals to remain in employment. Advice on fatigue management needs to be individualised, collaborative and address a broad range of factors in order to positively impact on work performance and participation [23].

The impact of fatigue on mood was discussed frequently in interviews. Participants described how when they are tired, they become frustrated with themselves and irritable with their colleagues. This then results in feelings of guilt, which increases their stress and that in-turn increases their fatigue. This could be due to many reasons such as pressures in work, fear of losing their job, and difficulty fulfilling roles outside of work. Fatigue and mood are closely related and can become an unending cycle without appropriate interventions. This is therefore an area that needs to be assessed during hospital outpatient appointments and referrals made to relevant health professionals to assist with managing feelings of guilt and stress.

### 4.2. Disclosure

The majority of interview participants had informed their employers of their diagnosis. They stated that they felt obliged to do this in order to explain time taken away from work to attend medical appointments. Most of the participants reported that their employers were supportive when informed and identified beneficial accommodations provided by employers. According to employment policy in Ireland, when an employee reports a disability, their employers are obliged to provide reasonable accommodations to enable them to participate fully in work [24]. This therefore indicates that some employers are meeting their responsibilities to employees with disabilities.

Some of the study participants, however, did not receive support or understanding from their colleagues about their disease, or their fatigue, on disclosure. They stated that fatigue is difficult to explain as it is not a visible symptom and that employers and co-workers do not understand or appreciate the difference between regular tiredness and the fatigue related to rheumatic diseases. This indicates a need for educating employers and employees about the nature of fatigue in rheumatic diseases, how it can impact work performance and recommended management strategies for the workplace. Fatigue management guidelines designed specifically for the workplace should focus on balancing work activities, correct positioning, work simplification strategies, stress and pain management, and effective communication training. This may improve productivity and perhaps reduce work absenteeism. Further research is required to examine the effectiveness of such work-based interventions on work ability.

Those who did not inform their employers explained this as being fearful of their employment being terminated or being disadvantaged for promotional purposes. Some of these interviewees were employed by private companies with one participant believing that there might be fewer disadvantages within other types of employment. However, employment legislation applies to employees regardless of category of employment and so when an employee receives less favourable treatment based on their disability, it is considered discrimination [24]. People with rheumatic diseases should therefore be informed of their right to fair treatment and reasonable accommodations in work to enable them to feel secure in their employment. The issue of how and when to disclose to employers was also discussed. Participants suggested input from health professionals on strategies for disclosing their disease to employers. This indicates that work-based self-management interventions should include effective communication strategies with employers and colleagues. 

### 4.3. Management of Fatigue in the Workplace

In order to manage the demands of work and fatigue, participants identified a range of management techniques including different types of energy conservation strategies and adapting a positive attitude. These approaches are commonly recommended by health professionals [25]. However, participants in this study reported that fatigue was often disregarded by their health professionals. Similarly Reeping-Wutts *et al.* [12] identified that fatigue is not routinely addressed by health professionals and that people with rheumatic diseases believe they have to accept fatigue as a consequence of their condition. This indicates the importance of multi-disciplinary rheumatology clinics with a range of health professionals, such as occupational therapists, who can advise on fatigue management. Participants of the current study discussed how they developed their own fatigue management strategies. Connolly *et al.* [26] also reported that fatigue management strategies were acquired through trial and error with no input from health professionals. This further supports the need for formal fatigue education for people with rheumatic diseases.

Some participants appeared to have developed maladaptive management strategies such as carrying out important work tasks at the beginning of the week, which resulted in severe fatigue by the end of the week. Similar findings on pushing through fatigue, and uncertainty on how to manage fatigue, have been identified in previous research [26]. In order to avoid this approach to fatigue management, Hewlett *et al.* [23] recommends that health professionals need to discuss fatigue as a legitimate and manageable symptom of rheumatic diseases.

## 5. Conclusions

Eighteen people with different types of rheumatic diseases participated in this study. All of them identified fatigue as the symptom of their disease that most interfered with their work. The participants described how fatigue impacted cognition, physical abilities and mood. Although this has been identified in other studies, the participants in this study also discussed specific work tasks affected by fatigue. These included reading documents, attending meetings, lifting and carrying objects in work, and remembering passwords for computers. Work-based management strategies were varied and mainly self-developed over time. This indicates a need for input from health professionals on work-based self-management interventions. These interventions could be carried out early in the disease trajectory and followed up with periodical input to manage the changing nature of rheumatic diseases.

The majority of study participants had informed their employers of their disease and had received support to assist them to manage their work. However, some participants discussed how, despite this, there was a lack of understanding from colleagues on the impact of fatigue and how it differs from regular tiredness. This indicates a need for formal education for employers and colleagues on the symptoms of rheumatic diseases with a particular focus on fatigue. 

A small number of participants had not informed their employers of their disease. Reasons for this related to fear with respect to job retention and reduced promotional prospects. Both of these reasons have financial implications and therefore could result in mental health issues for these participants. Self-management interventions, therefore, should include strategies on effective communication with employers for disclosure purposes and information on employee rights as identified in relevant employment legislation. 

Fatigue in rheumatic diseases is present in up to 90% of people [27]. This study has identified specific work tasks with which fatigue interferes. The study participants identified that their management strategies were mainly self-developed, indicating a need for formalized education on management strategies in the workplace. Further research is required to confirm the generalizability of these findings and to test the effectiveness of work-based fatigue management interventions.
